# Children’s Views About Their Future Career and Family Involvement: Associations With Children’s Gender Schemas and Parents’ Involvement in Work and Family Roles

**DOI:** 10.3389/fpsyg.2021.789764

**Published:** 2022-01-19

**Authors:** Joyce J. Endendijk, Christel M. Portengen

**Affiliations:** Child and Adolescent Studies, Utrecht University, Utrecht, Netherlands

**Keywords:** career, family, aspirations, gender identity, role models, parents, middle childhood

## Abstract

Substantial gender disparities in career advancement are still apparent, for instance in the gender pay gap, the overrepresentation of women in parttime work, and the underrepresentation of women in managerial positions. Regarding the developmental origins of these gender disparities, the current study examined whether children’s views about future career and family involvement were associated with children’s own gender schemas (gender stereotypes, gender identity) and parents’ career- and family-related gender roles. Participants were 142 Dutch families with a child between the ages of 6 and 12 years old (M = 9.80, SD = 1.48, 60% girls). The families had different compositions (1 parent, 2 parents, 1 to 3 children). Children completed a computer task assessing gender stereotypes about toys and questionnaires on gender identity (i.e., felt similarity to same- and other-gender children) and their views about future career and family involvement. Parents reported their occupation, work hours, and task division in the home, which were combined in a composite variable reflecting gender-typicality of career and family involvement. Generalized estimation equations were used to take into account dependency between family members. Results revealed that parents’, and especially mothers’, gender-typical career and family involvement was associated with children’s gender-typical views about future career and family involvement. In addition, children’s felt similarity to the same gender was associated with children’s gender-typical expectations about career and family involvement. These findings suggest that parents’ career, work hours, and task division in the home, together play an important role in how their children envision their future work and family roles. Children themselves also play an active role in developing this vision for the future by their own gender identity, specifically by how similar they feel to individuals of the same gender. To reduce gender disparities in the occupational and domestic domain, programs need to be designed that focus on parental role modeling in the family as well as children’s gender identity development.

## Introduction

Worldwide, substantial gender disparities in career advancement are still apparent, for instance in the gender pay gap (globally, women get paid approximately 20% less than men; International Labour Organization, 2019a), the overrepresentation of women in parttime work (25% of women compared to 10% of men, OECD, 2019), and the underrepresentation of women in managerial positions (<30%, International Labour Organization, 2019b). In addition, men’s involvement in the domestic sphere is clearly lacking behind the involvement of women (Croft et al., 2015). Men’s share of unpaid labor in the domestic sphere (i.e., childcare, household tasks) ranges from 22–38% in OECD countries (OECD, 2017b). Gender differences are also evident in the types of occupations men and women hold, with women being underrepresented in STEM fields (i.e., science, technology, engineering, mathematics) and men being underrepresented in fields such as health care and education (OECD, 2017a,b).

More gender equality in occupations, career advancement, and involvement in the domestic sphere is of utmost important for several reasons. First, a balanced engagement in both work and family roles is associated with increased general well-being in men and women (Grzywacz et al., 2008). Second, more gender-diversity in work teams improves team collaboration and performance (Bear and Woolley, 2011). Third, increased involvement of men in the domestic sphere reduces the burden on women, increases relationship satisfaction between partners (Stevens et al., 2001), and positively influences children’s cognitive development (Malin et al., 2014).

Early indications of gender differences in involvement with certain types of careers and family can be found in children’s gender-typical views about their future (Auger et al., 2005; Croft et al., 2014; Polavieja and Platt, 2014; Block et al., 2018). For instance, boys desired to become an athlete, mechanic, or soldier, whereas girls desired being an actor, hairdresser, or teacher (Polavieja and Platt, 2014). Girls also expected to be more family than career oriented in the future, whereas boys expected to be more career oriented than family oriented (i.e., gender-typical expectations about career and family involvement, Croft et al., 2014; Block et al., 2018). Importantly, longitudinal research shows that childhood career aspirations and expectations are linked over time with the actual attained careers of adults (Trice and McClellan, 1993; Mello, 2008; Lawson et al., 2018).

In order to further our understanding of gender disparities in involvement in career and domestic spheres, the current study examined whether children’s views about future career and family involvement were associated with children’s own gender schemas and parents’ gender-role behavior.

### Children’s Gender Stereotypes and Identity and Their Views About Future Career and Family Involvement

This research is based on predictions from gender-schema theories about factors in the child itself that might contribute to children’s gendered views about their future career and family involvement (Bem, 1981; Martin and Halverson, 1987). According to gender-schema theories children play an active role in their own gender development *via* their gender schemas. Gender schemas are dynamic cognitive structures containing gender-related information that children develop and actively construct based on their own experiences with gender in the social environment. Gender-schema theories also predict that gender schemas provide social standards that guide children’s behavior and choices. Therefore, based on gender-schema theories, we broadly expect that children with strong gender schemas hold more gender-typed views about their career and family life. Gender schemas encompass different types of schemas (e.g., gender stereotypes, gender attitudes, gender identity, gender self-concept), but the common element is that they concern how people think about themselves and each other in terms of gender (Tenenbaum and Leaper, 2002). The current study focuses on the following gender schemas: gender stereotypes and gender identity. Our specific expectations for these gender schemas are discussed below.

#### Children’s Gender Stereotypes

First, children’s gender stereotypes might play a role in children’s views about the future. There is ample evidence that children’s gender stereotypes about STEM (science, technology, engineering, mathematics) or intellectual ability are linked to gender-typical educational and career choices and interests (Steffens et al., 2010; Cheryan et al., 2015; Bian et al., 2017; Master et al., 2017). Another, less studied, gender-stereotype domain that is relevant to examine in relation to children’s views about their future career and family life is the domain of toys. Strong gender stereotypes about toys have been associated with more gender-typed toy play in children (Weisgram, 2016) and the degree of gender-typed play in preschool has been found to predict adolescents’ gender-typed occupational interests 10 years later (Kung, 2021). No studies have been done yet, that directly link children’s gender stereotypes about toys to gendered visions of their future selves (Fulcher and Coyle, 2018). However, the congruence principle of gender schema theories assumes that congruence exists between personal gender stereotypes and behaviors (Martin and Dinella, 2012). Based on this principle, one could expect strong gender stereotypes to be associated with children’s preference for gender-stereotyped occupations and the development of gender-stereotyped beliefs about children’s future career versus family involvement.

#### Children’s Gender Identity

Gender identity is a multidimensional construct, which has recently been conceptualized as involving both a connection to one’s own gender as well as to the other gender (Martin et al., 2017). The dual-identity conceptualization has been found to be particularly useful for describing individual differences in the (relative) extent to which children report to feel similar to peers of their own biological gender group and to peers of the opposite binary gender group. While most children feel stronger similarity with peers of their own gender than with peers of the other gender, i.e., report a *gender-typical identity* (Martin et al., 2017), the dual-identity approach also acknowledges experiences of transgender youth who feel dissimilar to peers of their own biological sex and more similar to peers of the opposite sex (Olson and Gülgöz, 2018). Differences in children’s felt (relative) similarity to own and other gender peers have been related to children’s social-emotional adjustment, gender-typed behavior, and gender attitudes (Andrews et al., 2016; Martin et al., 2017). A few studies in child and adult samples found that stronger gender identity typicality is associated with more traditional (i.e., gender-typed) occupational interests and career choices (Leaper and Van, 2008; Patterson, 2012; Dinella et al., 2014). An explanation for this congruence between gender identity and behavior/interests, is that children are motivated to make their behavior consistent with the behavior of the group they identify with (Martin and Dinella, 2012). This motivation is fueled by feelings of anxiety and discomfort when one violates the gender stereotypes and roles associated with one’s gender identity (Akerlof and Kranton, 2000). Further research is needed into how a dual-identity conceptualization of gender identity is associated with children’s views about their future career as well as family life.

### Parents as Models for Gender Roles

Social learning theories (Bandura, 1977; Bussey and Bandura, 1999) stress the importance of the social context in gender development. Central to these theories is the concept of observational learning and imitation of available models (especially same-gender models) in the child’s environment. In the family context, parents are models for future adult gender roles, for example, through the occupations they hold, the hours they work outside the home, as well as how they divide the tasks in the domestic sphere. By observing and imitating the differences between mothers and fathers in involvement with career and family, children will learn how males and females act which will shape their views about future career and family life. In addition, the modeling effect is supposed to be most likely for same-gender models because same-gender models provide information about what are appropriate behaviors for one’s own gender (Bandura, 1977). Children might therefore be more likely to model and internalize the occupations, work hours, and task division of same-gender parents in their anticipated career and family orientation.

Previous research indeed showed that different aspects of parents own career and family involvement (i.e., gender-typicality of occupation, work hours, task division at home) are associated with children’s views about future career and family involvement (Fulcher and Coyle, 2011; Croft et al., 2014; Polavieja and Platt, 2014; Platt and Polavieja, 2016; Oliveira et al., 2020). In general, these studies showed that more gender-typical engagement of parents with work and the family was associated with more gender-typical views about future career and family life in children. Yet, some studies did not find evidence for these associations (Fulcher, 2011; Croft et al., 2014). In addition, some studies demonstrated that the association between parents’ career and family involvement and children’s views about future career and family involvement was most salient in same-gender parent-child dyads (Fulcher and Coyle, 2011; Polavieja and Platt, 2014; Oliveira et al., 2020). Yet, there are also studies that did not find evidence for this same-gender modeling effect (Fulcher, 2011; Croft et al., 2014; Platt and Polavieja, 2016).

A possible explanation for the inconsistencies in previous research is that these studies did not examine the family as a system. Instead, in one study mother-child and father-child dyads were analyzed separately and siblings were treated as independent subjects (Croft et al., 2014). Other studies did not take into account dependency between mother and father in a family and/or focused on only one child in each family (Fulcher, 2011; Fulcher and Coyle, 2011; Polavieja and Platt, 2014; Oliveira et al., 2020). Such approaches fail to capture the richness of the family unit, reduce the overall statistical power as not all family members are included in a single analysis, and do not take into account dependency between family members. These issues can confound the effects that were found in previous research. The current study will therefore examine associations between children’s gendered views about future career and family involvement, children’s gender stereotypes and identity, and parents’ gender-role behaviors in the family as a whole.

### Middle Childhood as an Important Period for Studying Correlates of Children’s Views About Future Career and Family Involvement

Middle childhood (usually defined as ages 6—12) is an important, yet understudied, period for children’s gender development (Schroeder and Bámaca-Colbert, 2019) and specifically for children’s gendered views about the future. In middle childhood, there are some indications that parental factors appear to be stronger predictors of career aspirations than children’s own gender schemas (Croft et al., 2014) or personal attributes (Polavieja and Platt, 2014). Importantly, gender-typed views about future career and family life seem to become more gender-neutral toward the end of middle childhood and into adolescence, particularly for girls (Sandberg et al., 1991; Auger et al., 2005). In addition, in middle childhood children begin to develop gendered self-concepts, with boys seeing themselves as less communal and more agentic than girls (Block et al., 2018). The importance of gender as part of the self-concept appears to increase into early adolescence and reduces into later adolescence (Montemayor and Eisen, 1977). These gendered self-concepts could explain gender differences in anticipated prioritization of family over career in the future (Block et al., 2018), enrollment in male- and female-dominated high-school programs (Tellhed et al., 2018), and choices for STEM careers (Eccles and Wang, 2016). Moreover, children’s gender stereotypes increase between age 3 to 5 (Halim et al., 2013), peak between age 5 to 7, and become more flexible during middle childhood (Trautner et al., 2005) and flexibility continues to develop into adolescence (Bartini, 2006). Finally, after being able to identify one’s own gender around age 3, and an understanding of gender constancy at 6–7 years of age, in middle childhood children develop a more complex and multidimensional gender identity (Halim and Ruble, 2010). All these developments make middle childhood an appealing setting for studying predictors of children’s gendered views about their future life.

### Current Study: Research Questions and Hypotheses

In sum, the current study employed a family-systems approach to examine child and family correlates of children’s (6–12-year-old) views about their future career and family involvement. Correlates at the child level consisted of gender stereotypes about toys and gender identity. Correlates at the family level consisted of the gender-typicality of mothers’ and fathers’ occupation (i.e., the proportion of same-gender individuals that work in a certain occupational domain), work hours (i.e., mothers working parttime, fathers working full-time), and task division at home (i.e., degree to which mothers are more responsible for household and child-care tasks than fathers). The following hypotheses were tested:

(1) Children with strong gender schemas (i.e., traditional gender stereotypes about toys, gender-typical identity) hold more gender-typical views about one’s future career and family involvement.
(2) Gender-typicality of parents’ occupations, work hours, and task division at home is associated with more gender-typical views about future career and family involvement of children.
(3) Associations between gender-typicality of parents’ occupations, work hours, and task division at home on the one hand, and children’s gender-typical views about career and family involvement on the other hand, are more salient in same-gender dyads than in mixed-gender dyads.

## Materials and Methods

### Participants

Student assistants (BA and MA students in Clinical, Child, Family, and Education studies at Utrecht University) used their personal networks to recruit Dutch families with at least one child between the ages of 6 and 12 years old for this study. Families were contacted *via* information letters (provided in-person or *via* e-mail). The student assistants recruited 142 families. Recruitment and data collection took place between September 2018 and June 2021. The only exclusion criterion was not being able to understand or read Dutch instructions.

From each participating family, one parent (*n* = 36) or two parents (*n* = 106) participated. In total, 139 mothers and 108 fathers participated. Regarding the number of participating children per family, in about half of the families (55%, *n* = 78) only one child was between the ages of 6–12. In 42% (*n* = 60) of families two children were in the target age range, and in 3% (*n* = 4) of families three children were in the target age range. Table 1 presents the background characteristics of this sample. Generally, the majority of the parents in the sample were highly educated.

**TABLE 1:** Sample characteristics.

Family characteristics	
Number of children, range (*M*)	1–5 (2.35)
**Gender composition of children, *n* (%)**	
All girls	26 (18)
All boys	33 (23)
Mixed gender composition	83 (59)
**Family composition, *n* (%)**	
Heterosexual two-parent family	127 (90)
Single parent or divorced	15 (10)
**Child characteristics**	
Age, *M* (*SD*)	9.80 (1.48)
Female gender, *n* (%)	125 (60)
**Mothers’ characteristics**	
Age, *M* (*SD*)	42.44 (4.92)
**Educational level, *n* (%)*^a^***	
Primary education	1 (1)
Lower secondary education	10 (7)
Higher secondary education	40 (29)
Higher vocational education	47 (34)
University	41 (29)
**Fathers’ characteristics**	
Age, *M* (*SD*)	44.42 (5.16)
**Educational level, *n* (%)*^a^***	
Primary education	–
Lower secondary education	8 (7)
Higher secondary education	30 (28)
Higher vocational education	38 (35)
University	32 (30)

*^a^Educational levels are sorted from lowest to highest level.*

### Procedure

Families were visited at their home by the student assistant who recruited the family. Participants provided written informed consent for their participation at the beginning of the home visit. Each family member subsequently completed questionnaires and a computer task (see section “Instruments”) *via* LimeSurvey on a laptop or desktop (duration: approximately 15 min). Parents completed the questionnaires and computer task independently by following the instructions that were presented to them in the LimeSurvey environment. Children completed the questionnaires and computer task under supervision of the student assistant who gave the child verbal instructions. Families received no compensation for their participation. The Ethics Committee of the Faculty of Social Sciences at Utrecht University approved the study (number FETC18-097).

### Instruments

#### Children’s Views About Future Career and Family Involvement

Two aspects of children’s views about one’s future career and family involvement were measured. First, to assess the gender-typicality of children’s desired future career, children were asked the following question: “What do you want to be when you grow up?” Children’s free responses to this question were coded for gender-typicality of the desired career/occupation. Therefore, we used Dutch Central Bureau of Statistics (CBS), 2021 data that provides information on the proportion of men and women in an extensive list of occupations. The proportion of women or men (depending on the child’s gender) in a certain occupation that corresponded with the occupation mentioned by the child was used for our analyses. Higher scores (>0.50) indicated more gender-typicality of a certain occupation, lower score (<0.50) represent more gender-atypical occupations. When children indicated multiple occupations, the proportions were averaged. In case children answered the question with “I don’t know” (or something similar) a proportion of 0.5 (i.e., neutral score) was used for this child.

Second, to assess children’s expectations about relative future involvement with career versus family, children were presented with two own-gender individuals and a description of their career and family life (Croft et al., 2014, see Supplementary Figure 1 for an example). For each pair of individuals (i.e., two pairs were used), a person who worked full time was contrasted with a person who stayed at home caring for the children. Children were asked to indicate for each pair of individuals who they think they will be more like when they are grown up. They rated their similarity on a 5-point scale (1 = most similar to career-oriented target, 2 = a bit more similar to the career-oriented target, 3 = equally similar to both targets, 4 = a bit more similar to the family-oriented target, 5 = most similar to the family-oriented target). Scores were recoded separately for boys and girls and averaged over the two items, in such a way that higher average scores indicated gender-typical expectations of family versus career involvement (i.e., for boys more involvement with career than with family, for girls more involvement with family than with career).

#### Child Gender Stereotypes About Toys

Children completed a computer task (action inference paradigm; Endendijk et al., 2013) to assess gender stereotypes about toys. The validity of this task to assess gender stereotypes about toys in parents and children has been demonstrated (Endendijk et al., 2013). Participants were asked to divide toys (see Supplementary Table 1 for a list of toys used) between two fictitious children as quickly as possible, by means of pressing one of two keys on the keyboard (“e” or “i”) that were assigned to each child. Pictures of the two children (full color) were presented constantly in the left- and right-hand upper corners of the computer screen. Each full-color toy was presented in the middle of the screen until the participant hit the response key, after which the next full-color toy emerged on the screen.

The task started with a practice block (20 trials) in which red and blue presents had to be divided between two gender-neutral children (could be labeled as both a boy or a girl), followed by two stereotype-congruent blocks and two stereotype-incongruent blocks (17 trials in each block). In the congruent blocks, participants were instructed to assign stereotypically feminine toys (e.g., doll) to a girl and stereotypically masculine toys (e.g., car) to a boy. In the incongruent blocks, participants were instructed to assign stereotypically feminine toys to a boy and stereotypically masculine toys to a girl. To reduce order effects of the presentation of congruent and incongruent blocks (Nosek et al., 2005), the two congruent blocks alternated with the two incongruent blocks (i.e., congruent-incongruent-congruent-incongruent) so that participants made each possible switch between congruent and incongruent blocks. Participants were given a rest period between each block of self-determined length (instructions for the next block were provided in this rest period as well).

The Action Inference Paradigm (AIP) is similar in design to the widely used Implicit Association Test (IAT), as in both tasks a prepotent response tendency (e.g., sort stimuli in a stereotype-consistent way) may either facilitate or interfere with the response required in the task, which in turn influences the speed and accuracy of participants’ responses. The main difference between the IAT and AIP is that in the AIP participants must sort only one type of stimuli (e.g., toys) between two categories, whereas in IATs two types of stimuli (e.g., concepts, such as male and female names, and attributes, such as career and family words) must be sorted between two categories, which might be more difficult for children.

The improved scoring algorithm of Greenwald et al. (2003) was used to determine the level of gender stereotypes of the participant. More details about the scoring can be found in the Supplementary Material. In short, the gender stereotype score calculated with this algorithm reflects the difference in response latencies between stereotype-incongruent blocks and stereotype-congruent blocks (divided by the pooled SD of response latencies across all trials). Higher scores indicate stronger stereotypical ideas about the appropriateness of certain toys for girls and boys.

#### Child Gender Identity

Children completed a dual gender identity questionnaire developed and validated by Martin et al. (2017). Participants answered 10 questions regarding how similar they felt to both boys and girls (e.g., “How similar do you feel to [boys/girls]?”) using a graphical response scale with circles indicating the level of similarity. Participants answered questions about similarity in five domains: general similarity, behavior, appearance, activities, spending time together. Responses ranged from 0 (circles farthest apart) to 4 (overlapping circles). Separate composite scores were created for the 5 items reflecting similarity to the same-gender group and for the 5 items reflecting similarity to the other-gender group. Higher average scores on these scales reflect more similarity. Reliability of the two scales was good (Cronbach’s alpha = 0.85, 0.82, for respectively same-gender and other-gender similarity).

#### Gender-Typicality of Parents Career and Family Involvement

##### Parents’ Occupation

Parents were asked to report their current occupation. Their responses to this question were coded for gender-typicality the same way as we coded children’s aspired occupations, by using CBS data. Twenty percent (*n* = 100) of the total number of reported careers by parents and children were double coded independently by the first and second authors. The intraclass correlation coefficient (ICC = 0.90) demonstrated excellent coder reliability across this subset of careers. The first author coded the remainder of the careers.

##### Parents’ Work Hours

Parents were asked to report their working hours (i.e., for paid work) per week. Mothers’ work hours were inversed (maximum work hours of mothers in this sample subtracted from each mother’s work hours) so that higher scores represented more traditional work behaviors (i.e., working less hours outside the house).

##### Parents’ Task Division in the Home

Parents filled out a 15-item questionnaire on their perception of the division of labor regarding small household tasks (e.g., buying groceries, cooking dinner, cleaning) and child-care tasks (e.g., bring children to bed, bathe children, bring children to school) during the past week (Endendijk et al., 2018). Parents could answer on a five-point scale (1 = I exclusively/almost exclusively performed this task, 5 = my partner exclusively/almost exclusively performed this task). Scores on the 15 items were recoded and averaged in such a way that mean scores around 3 represent an egalitarian task division, scores above 3 represent more maternal involvement in the family and scores below 3 represent more paternal involvement in the family. Reliability of this scale in the current study was good (Cronbach’s alpha = 0.86). Single parents (*n* = 6) were also asked to complete this questionnaire. There was variation in their mean scores (range = 1.67–5.00). We checked whether exclusion of these families influenced our results, but this was not the case. Therefore, we decided to keep these families in our sample.

##### Creation of Composite Gender-Typicality Variable

Following Fulcher and Coyle (2011), we combined gender-typicality of parents’ occupation, work hours, and task division in one aggregate variable. First, scores on each variable were recoded in such a way that higher scores reflected more gender-typicality (e.g., more gender-typical occupation, higher paternal work hours, lower maternal work hours, more maternal involvement with small-household and child-care tasks). Second, recoded scores for each variable were standardized into Z-scores and subsequently averaged to create a variable reflecting gender-typicality of parents’ career and family involvement. This approach would reduce the number of predictors entered in further analyses. Results of analyses on separate career (work hours, occupation) and family (task division) variables are presented in the Supplementary Material (Supplementary Tables 2, 3, effects are in the same direction but are no longer significant, *p*-values between 0.105 and 0.173).

### Analyses

Generalized Estimating Equations (GEE) were used to analyze the data (Homish et al., 2010) in SPSS (version 24). GEE models are regression-based models that can take into account dependency between variables, such as in family data (Homish et al., 2010). GEE models are more flexible for missing data compared to other models (Zeger et al., 1988) and are therefore suitable for our family data with different family compositions. In addition, in case of a small number of observations in each cluster (i.e., sparse data) GEE is a more robust alternative to multilevel modeling (McNeish, 2014). The family data in the current study could be considered as sparse data because the number of observations per family (i.e., family members) ranged from two to five. Other advantages of GEE over multilevel models include easier model computation and interpretation, more robustness to model misspecification, and no need to model random effects that are not of interest for the research question (McNeish et al., 2017). GEE has been applied to analyze family data in samples ranging from as small as 47 families (Abraham et al., 2021) up to 191 families (Rossen et al., 2018).

Two separate GEEs were conducted, one for children’s desired future career and one for children’s expectations about future career and family involvement. Each model included main effects for children’s gender stereotypes and gender identity (similarity to same- and other gender), and gender-typicality of parents’ career and family involvement. In addition, we added a two-way interaction between parent gender and gender-typicality of parents’ career and family involvement. This allowed for testing whether associations between gender-typicality of parents’ career and family involvement and children’s views about future career and family involvement were driven primarily by mothers or fathers. Finally, we added a two-way interaction between gender-composition of the parent-child dyad (same-gender vs. mixed-gender) and gender-typicality of parents’ career and family involvement. This enabled testing whether the associations between gender-typicality of parents’ career and family involvement and children’s views about future career and family involvement were stronger for same-gender dyads. The GEE models were specified with a Gaussian distribution with an identity link for each family, as the dependent variables were continuous (Homish et al., 2010). An exchangeable correlation structure was considered to be most appropriate for the family data (Homish et al., 2010; McNeish et al., 2017). Robust standard errors (Hubert/White Sandwich Estimators) were computed to ensure valid estimations even in case of a mis-specified correlation structure. Parameter estimates were presented as regression coefficients, so that the analyses could be interpreted the same as general linear regression models. For each analysis, we determined which covariates needed to be included based on the change-in-estimate method, >5% change criterion (Rothman et al., 2008).

## Results

### Descriptive Statistics

Tables 2, 3 display descriptive statistics and correlations for all study variables, for children and parents separately. All variables approached a normal distribution. Several outliers were identified (gender stereotypes: *n* = 2, other-gender similarity: *n* = 2, task division: *n* = 2). These outliers were winsorized (highest non-outlying number + difference between highest non-outlying number and before highest non-outlying number; Tabachnick and Fidell, 2012).

**TABLE 2:** Descriptive statistics of child study variables.

	1.	2.	3.	4.	*M* (*SD*)
1. Gender-typical desired future career					0.59 (0.20)
2. Gender-typical expectation about career-family	0.10				3.11 (0.84)
3. Gender stereotypes about toys	0.00	−0.08			0.22 (0.33)
4. Same-gender similarity	0.07	0.19**	0.04		4.00 (0.88)
5. Other-gender similarity	−0.07	0.03	−0.15*	−0.54**	2.11 (0.79)

*p < 0.05; **p < 0.01.

**TABLE 3:** Descriptive statistics of parent study variables.

				Mothers	Fathers
				
	1.	2.	3.	*M* (*SD*)	*M* (*SD*)
1. Gender-typical career		−0.23**	0.09	0.63 (0.20)	0.68 (0.23)
2. Work hours	0.36**		−0.32**	25.81 (9.81)	38.51 (10.16)
3. Gender-typical task division	0.24*	−0.29**		3.79 (0.58)	3.51 (0.64)

*Correlations above the diagonal are for mothers. Correlations below the diagonal are for fathers.*

*p < 0.05; **p < 0.01.

As can be seen in Table 2, for children, same-gender similarity was significantly associated with more gender-typical expectations about future career versus family involvement. Same-gender similarity was negatively associated with other-gender similarity. More other-gender similarity was associated with less strong gender stereotypes about toys. None of the other child variables were significantly correlated. Independent *t*-tests were conducted to examine gender differences on the child variables. First, the proportion of women in girls’ desired careers (*M* = 0.59, *SD* = 0.20) was significantly higher than the proportion of women in boys’ desired careers [*M* = 0.41, *SD* = 0.20, *t*(208) = 6.54, *p* < 0.001]. Boys were thus more likely to desire careers in which men were overrepresented, whereas girls were more likely to desire careers in which women were overrepresented. Second, girls expected more gender-typical family versus career involvement (*M* = 3.29, *SD* = 0.82) than boys [*M* = 2.85, *SD* = 0.79, *t*(208) = 3.85, *p* < 0.001]. Girls also reported more other-gender similarity (*M* = 2.28, *SD* = 0.76) than boys did [*M* = 1.88, *SD* = 0.77, *t*(208) = 3.70, *p* < 0.001]. However, boys reported more same-gender similarity (*M* = 4.16, *SD* = 0.76) than girls did [*M* = 3.88, *SD* = 0.94, *t*(201.04) = −2.38, *p* = 0.018]. There was no gender difference in children’s gender stereotypes [*t*(208) = −0.81, *p* = 0.418] (i.e., boys and girls did not differ in response latencies to stereotype-inconsistent versus stereotype-consistent trials in the task assessing gender stereotypes about toys).

As can be seen in Table 3, for fathers, there were significant associations in the expected direction between work hours, task division, and gender-typicality of their career. For mothers, more work hours were associated with a less traditional task division as well as a less gender-typical career. An independent *t*-test on the proportion of women in the occupations that fathers and mothers reported themselves to be in, revealed that mothers reported occupations with a higher proportion of women (*M* = 0.63, *SD* = 0.20) than fathers [*M* = 0.32, *SD* = 0.23, t(208,76) = 11.28, *p* < 0.001].

As all the correlations between the independent variables in Tables 2, 3 were below 0.70, there were no issues with multi-collinearity in further analyses.

### Predictors of the Gender-Typicality of Children’s Desired Future Career

Table 4 displays results for the final GEE model for children’s gender-typical desired future career. Only parents’ gender-typical career and family involvement was associated with the gender-typicality of children’s desired career. Children’s gender stereotypes about toys and same- and other-gender similarity were not related to children’s desired career. Regarding the covariates, younger child age and older parental age were associated with more gender-typical desired careers.

**TABLE 4:** Generalized estimation equations predicting gender-typicality of children’s desired career from children’s gender identity, stereotypes, and parents’ gender-typical career and family involvement.

	*B*	*SE*	95% CI	Wald	*p*
Child gender^1^	0.01	0.04	[−0.06, 0.08]	0.11	0.741
Child age	−0.03*	0.01	[−0.05, −0.004]	5.65	0.017
Parent gender^2^	0.01	0.01	[−0.003, 0.02]	2.11	0.146
Parent age	0.004*	0.002	[0.00, 0.01]	4.09	0.043
Educational level^3^					
Primary education	−0.003	0.04	[−0.08, 0.08]	0.01	0.934
Lower secondary education	0.02	0.05	[−0.08, 0.13]	0.18	0.676
Higher secondary education	−0.06	0.04	[−0.13, 0.01]	2.68	0.101
Higher vocational education	−0.02	0.04	[−0.09, 0.06]	0.19	0.661
Family composition^4^					
Single parent/divorced	0.05	0.03	[−0.01, 0.12]	2.38	0.123
Child gender stereotypes about toys	−0.01	0.04	[−0.10, 0.08]	0.05	0.818
Child same-gender similarity	0.01	0.02	[−0.04, 0.06]	0.17	0.679
Child other-gender similarity	−0.01	0.02	[−0.05, 0.03]	0.19	0.663
Gender-typicality of parents’ career and family involvement^5^	0.03*	0.01	[0.003, 0.05]	4.94	0.026

*^1^Boys are reference category.*

*^2^Fathers are reference category.*

*^3^University level was the reference category.*

*^4^Two-parent family was the reference category.*

*^5^This variable is a standardized composite score including gender-typicality of work hours, gender-typicality of occupation, and gender-typicality of task division.*

*p < 0.05.

The additional interaction between parent gender and parents’ gender-typical career-family involvement was not significant (*B* = −0.001, *SE* = 0.01, 95% CI = −0.02, 0.02, Wald = 0.002, *p* = 0.962). This indicated that the association between parents’ gender-typical career-family involvement and children’s desired future career was not driven primarily by mothers or fathers. The additional interaction between gender composition of the parent-child dyad and parents’ gender-typical career-family involvement was not significant (*B* = −0.01, *SE* = 0.02, 95% CI = −0.04, 0.02, Wald = 0.15, *p* = 0.704). This indicated that the association between parents’ gender-typical career-family involvement and children’s desired career was not different for same-gender and other-gender parent-child dyads.

### Predictors of the Gender-Typicality of Children’s Expectations About Future Career and Family Involvement

Table 5 displays results for the final GEE model for children’s gender-typical expectations about involvement with career and family. More same-gender similarity in children was associated with more gender-typical expectations about involvement with career and family. Children’s gender stereotypes about toys and other-gender similarity were not related to children’s gender-typical career-family expectations. Regarding the covariates, being a girl and younger child age were associated with more gender-typical expectations about future career and family involvement.

**TABLE 5:** Generalized estimation equations predicting children’s gender-typical expectations about future career and family involvement from children’s gender identity, stereotypes and parents’ gender-typical career and family involvement.

	*B*	*SE*	95% *CI*	Wald	*p*
Child gender^1^	0.38*	0.14	[0.12, 0.65]	7.93	0.005
Child age	−0.11*	0.04	[−0.18, −0.04]	8.49	0.004
Parent gender^2^	−0.03	0.03	[−0.09, 0.03]	1.03	0.310
Parent age	−0.01	0.01	[−0.03, 0.01]	0.50	0.482
Family gender composition^3^					
All boys	−0.08	0.17	[−0.41, 0.25]	0.22	0.641
All girls	0.03	0.15	[−0.26, 0.33]	0.47	0.828
Child gender stereotypes about toys	−0.27	0.16	[−0.59, 0.04]	2.92	0.087
Child same-gender similarity	0.24*	0.08	[0.07, 0.40]	8.17	0.004
Child other-gender similarity	0.11	0.08	[−0.05, 0.28]	1.78	0.182
Gender-typicality of parents’ career and family involvement	−0.09	0.06	[−0.21, 0.03]	2.12	0.146
Parent gender*Gender-typicality career-family involvement^2^	0.10*	0.05	[0.01, 0.19]	4.49	0.034

*^1^Boys are reference category.*

*^2^Fathers are reference category. This variable is a standardized composite score including gender-typicality of work hours, gender-typicality of occupation, and gender-typicality of task division.*

*^3^Mixed gender composition of children is the reference category.*

*p < 0.05.

In addition, the interaction between parent gender and parents’ gender-typical career-family involvement was significant (*B* = 0.10, *SE* = 0.05, 95% CI = 0.01, 0.19, Wald = 4.49, *p* = 0.034). This indicated that only mothers’ gender-typical career and family involvement was associated with children’s gender-typical expectations about future career and family involvement.

The additional interaction between gender composition of the parent-child dyad and parents’ gender-typical career-family involvement was not significant (*B* = −0.05, *SE* = 0.07, 95% CI = −0.19, 0.10, Wald = 0.38, *p* = 0.536). This indicated that the association between parents’ gender-typical career-family involvement and children’s expected career-family involvement was not different for same-gender and mixed-gender parent-child dyads.

## Discussion

This study was conducted to examine whether children’s views about future career and family involvement were associated with children’s own gender stereotypes and identity as well as parents’ gender-typical career and family involvement. Results revealed that parents’, and especially mothers’, gender-typical career and family involvement was associated with children’s gender-typical views about their future career and family life. In addition, children’s felt similarity to the same gender as well as mothers’ gender-typical career and family involvement were associated with children’s gender-typical expectations about their future career and family involvement. Children’s gender stereotypes about toys were not related to children’s views about future career and family involvement. Finally, associations between parent’s gender-typical career and family involvement and children’s views about their future were not different between same-gender and mixed-gender parent-child dyads.

Our findings for children’s gender identity provide some support for gender-schema theories’ prediction that gender schemas provide social standards that guide children’s behavior and choices (Bem, 1981; Martin and Halverson, 1987). Children with strong gender schemas, for example because they felt high similarity to same-gender peers, in this study indeed held more gender-typical expectations about future career and family involvement but did not desire a more gender-typical career. An explanation for the congruence between the level of same-gender similarity and children’s gender-typical expectations about their career and family involvement, is that children are motivated to make their behavior consistent with the behavior of the group they identify with (Martin and Dinella, 2012). That desired career was not linked with children’s gender identity might be because children in middle childhood children still have limited knowledge of the gender typicality of occupations (Gottfredson, 2002). Our findings extend previous research linking higher gender-typicality to more traditional occupational interests and career choices (Leaper and Van, 2008; Patterson, 2012; Dinella et al., 2014), by showing that gender identity aspects also relate to expectations about future involvement in the domestic sphere.

Unexpectedly, children’s gender stereotypes, specifically in relation to toys, were not related to their views about their future career and family involvement. It could be that the link between children’s gender stereotypes about toys and children’s views about their future is too indirect to be found without also examining possible underlying mediating factors. For instance, gender stereotypes about toys have been associated with gender-typed toy play (Weisgram, 2016) which in turn has been associated with adolescents’ gender-typical occupational interests (Kung, 2021). Future research could examine this mediational process. The lack of associations with children’s gender stereotypes about toys might also be due to our measure including both toys that have a clear link with the domestic sphere (e.g., baby dolls, toy kitchen) or the career sphere (e.g., fire truck, tools), as well as toys that are less directly linked to these domains (e.g., pirate costume, princess costume). Our measure consisted of too few trials to examine the effect of toy type. Future research could examine whether children’s gender stereotypes about toys with clear links to the career or domestic spheres are related to their views about future career and family involvement.

We also found some evidence for the role modeling prediction from social learning theory (Bandura, 1977; Bussey and Bandura, 1999). It appears that parents’, and especially mothers’, gender-typical career and family involvement are associated with children’s views about future career and family life. Our findings demonstrate that previously found associations between parents’ work- and family-related gender roles and children’s career and family aspirations (Fulcher and Coyle, 2011; Croft et al., 2014; Polavieja and Platt, 2014; Oliveira et al., 2020) also hold in a family-systems context. In the current study parents could provide a model for traditional gender-role behavior by working in a career domain with a high percentage of same-gender peers, when mothers worked few hours outside the home, when fathers worked many hours outside the home, and when mothers were more responsible than fathers for household and childcare tasks. By observing such traditional gender roles in the career *and* family involvement of their parents, children will learn how males and females act, which will shape their views about their future career and family life. An explanation for why especially mothers’ work- and family-related gender roles were important for children’s expectations about career versus family involvement could be that especially mothers might provide a model for balancing work and family roles. Indeed, mothers have been found to experience more work-family conflict than fathers (Shockley et al., 2017).

No support was found for the same-gender modeling hypothesis of social learning theory (Bandura, 1977; Bussey and Bandura, 1999). Previous research also produced mixed findings regarding same-gender modeling of parents’ career and family involvement (Fulcher, 2011; Fulcher and Coyle, 2011; Croft et al., 2014; Polavieja and Platt, 2014; Oliveira et al., 2020). In the current study the associations between parents’ gender-typical career and family involvement and children’s views about future career and family involvement, were not more salient in same-gender dyads than in mixed-gender dyads. It appears that fathers and mothers are important role models for both boys and girls. This might not be surprising as mothers’ and fathers’ gender roles in a family are closely interrelated (Oláh and Neyer, 2021). For instance, when one parent increases their work hours, the other parent is likely to compensate for the reduced involvement in the family (Hook, 2006; Fox, 2009). So, it might actually be the combination of mothers and fathers work in and outside the family that conveys messages to children about how men and women balance work and family responsibilities and that shapes children’s views about future career and family life.

A final noteworthy finding is that we found correlational evidence for a possible developmental process implicated in children’s views about their future selves, as older child age was associated with less gender-typical views about career and family. This finding fits with previous research demonstrating that children’s gender stereotypes become more flexible and less rigid over time (Trautner et al., 2005). In addition, this finding is noteworthy because it could imply that children’s views about their future career and family involvement might over time become less congruent with their gender identity or their parents’ career and family involvement (assuming that the latter two factors remain relatively constant over time). This hypothesis remains to be tested longitudinally, as well as how children experience or resolve this increasing incongruence.

Even though our study is strong in terms of the family-systems approach and the use of mixed methods (i.e., computer task, parent-report, child-report), our findings must be viewed in light of some limitations. First, because of the correlational design of this study, we were not able to determine the direction of effects in the association that were found. More longitudinal research is now necessary to unravel the gendered developmental processes underlying the career decision making process. Second, our sample size was too small to optimally utilize the dual gender identity approach by examining how different gender-identity typologies are related to children’s gender-typical views about career and family involvement. Third, even though we, and previous studies (e.g., Croft et al., 2014), found relevant associations with children’s expected future involvement with career and family, the measure used to assess children’s expectations only consisted of 2 items. Future research could extend this measure to assess children’s gendered expectations for the future in a more multi-faceted way. Finally, a convenience sampling method was used, which resulted in a sample that was more highly educated than the population.

In sum, this family-systems study demonstrated that parents’ own career, work hours, and task division in the home, together play an important role in how their children envision their future work and family roles. This suggests that intergenerational transmission plays a role in the perpetuation of gender disparities in the occupational and domestic domain. Children themselves also play an active role in developing this vision for the future by their own gender identity, specifically by how similar they feel to individuals of the same gender. A practical implication of these findings is that parents need to be made aware of the roles their own gender-role behavior, as well as their children’s gender identity, play in the career decision making process of their children. For boys and girls to make career decisions that fit with their interests and competencies, instead of their gender or their parents’ gender roles, parents could encourage children to explore a wide range of career and educational options. In addition, programs and policies could stimulate more equality in parental gender roles as well as children’s felt similarity to people of both genders, in order to reduce gender disparities in the occupational and domestic domain.

## Data Availability Statement

The raw data supporting the conclusions of this article will be made available by the authors, without undue reservation.

## Ethics Statement

The studies involving human participants were reviewed and approved by The Ethics Committee of the Faculty of Social Sciences at Utrecht University (number FETC18-097). Written informed consent to participate in this study was provided by the participants’ legal guardian/next of kin.

## Author Contributions

JE: conceptualization, formal analysis, methodology and design, and writing – original draft. JE and CP: supervision of data collection and processing of data. CP: writing – review and editing. Both authors contributed to the article and approved the submitted version.

## Conflict of Interest

The authors declare that the research was conducted in the absence of any commercial or financial relationships that could be construed as a potential conflict of interest.

## Publisher’s Note

All claims expressed in this article are solely those of the authors and do not necessarily represent those of their affiliated organizations, or those of the publisher, the editors and the reviewers. Any product that may be evaluated in this article, or claim that may be made by its manufacturer, is not guaranteed or endorsed by the publisher.
