# Supplementary material for: Children’s Views About Their Future Career and Family Involvement: Associations With Children’s Gender Schemas and Parents’ Involvement in Work and Family Roles
Source: Front Psychol. 2022 Jan 19;12:789764. doi: 10.3389/fpsyg.2021.789764 (PMC8809201; doi:10.3389/fpsyg.2021.789764)
Supplement: Supplementary file 1 [file Data_Sheet_1.docx]

**Supplementary Material**

**Table S1**

*Toys Used in the Gender Stereotypes Computer Task*

| Feminine toys | Masculine toys |
| --- | --- |
| Doll clothes (2x) | Tractor |
| Doll bath | Crane |
| Doll with horse | Race car |
| Baby doll (2x) | Toy body shop |
| Doll in pram | Tool set (2x) |
| Toy kitchen | Construction toys |
| Stroller | Ball |
| Crib | Train |
| Doll house | Power shovel |
| Barbie with kitchen | Fire truck |
| Barbie | Motorbike |
| Baby doll with accessories | Airplane (2x) |
| Princess doll | Helicopter |
| Princess costume | Pirate costume |
| Hoolahoop | Skateboard |

**Details of the Improved Scoring Algorithm Used to Calculate Children’s Gender Stereotype Scores**

Step 1: Accuracy and reaction time (RT) data from two congruent and two incongruent blocks are used.

Step 2: Eliminate trials with RTs > 10,000 ms. Eliminate subjects for whom more than 10% of trials have RTs less than 300 ms (this was not necessary in our sample).

Step 3: Use all remaining trials.

Step 4: Compute mean of correct RTs for each block.

Step 5: Compute one pooled SD for all trials in the first congruent and incongruent blocks; compute another pooled SD for all trials in the second congruent and incongruent blocks.

Step 6: Compute an error RT by adding 600ms to the block means from stem 4. In each trial in which an error was made, replace the RT with the error RT.

Step 7: Compute average RT for each block of trials.

Step 8: Compute two difference scores: first incongruent block RT – first congruent block RT, second incongruent block RT – second congruent block RT.

Step 9: Divide each difference by its associated pooled SD from Step 5.

Step 10: Average the two quotients from Step 9.

**Table S2**

*Generalized Estimation Equations Predicting Gender-Typicality of Children’s Desired Career From Children’s Gender Identity, Stereotypes, and Parents’ Gender-Typical Career and Family Involvement*

|  | *B* | *SE* | 95% *CI* | Wald | *p* |
| --- | --- | --- | --- | --- | --- |
| Child gender^1^ | .01 | .04 | [-.06, .08] | 0.12 | .734 |
| Child age | -.03* | .01 | [-.05, -.004] | 5.60 | .018 |
| Parent gender^2^ | .01 | .01 | [-.004, .02] | 2.05 | .152 |
| Parent age | .004 | .002 | [.00, .01] | 3.48 | .062 |
| Educational level^3^ |  |  |  |  |  |
| Primary education | -.01 | .04 | [-.10, .07] | 0.10 | .754 |
| Lower secondary education | .02 | .05 | [-.09, .13] | 0.14 | .706 |
| Higher secondary education | -.06 | .04 | [-.13, .01] | 2.65 | .104 |
| Higher vocational education | -.02 | .04 | [-.09, .06] | 0.18 | .672 |
| Family composition^4^ |  |  |  |  |  |
| Single-parent/divorced | .06 | .04 | [-.01, .12] | 2.43 | .119 |
| Child gender stereotypes about toys | -.01 | .04 | [-.10, .07] | 0.06 | .802 |
| Child same-gender similarity | .01 | .02 | [-.04, .06] | 0.17 | .678 |
| Child other-gender similarity | -.01 | .02 | [-.05, .03] | 0.19 | .665 |
| Gender-typicality of parents’ career | .002 | .01 | [-.01, .01] | 0.13 | .718 |
| Gender-typicality of work hours | .01 | .01 | [-.003, .03] | 2.44 | .119 |
| Gender-typicality of task division | .01 | .01 | [-.003, .03] | 2.58 | .108 |

^1^ Boys are reference category.

^2^ Fathers are reference category.

^3^ University level was the reference category.

^4^ Two-parent family was the reference category.

^5^ This variable is a standardized composite score including gender-typicality of work hours and gender-typicality of occupation.

* *p* < .05

**Table S3**

*Generalized Estimation Equations Predicting Children’s Gender-Typical Expectations About Future Career and Family Involvement From Children’s Gender Identity, Stereotypes, and Parents’ Gender-Typical Career and Family Involvement*

|  | *B* | *SE* | 95% *CI* | Wald | *p* |
| --- | --- | --- | --- | --- | --- |
| Child gender^1^ | .37* | .14 | [.11, .64] | 7.67 | .006 |
| Child age | -.12* | .04 | [-.19, -.04] | 8.77 | .003 |
| Parent gender^2^ | -.03 | .03 | [-.09, .03] | 0.84 | .358 |
| Parent age | -.01 | .01 | [-.02, .01] | 0.24 | .622 |
| Family gender composition^3^ |  |  |  |  |  |
| All boys | -.07 | .17 | [-.40, .26] | 0.19 | .667 |
| All girls | .04 | .15 | [-.26, .33] | 0.06 | .800 |
| Child gender stereotypes about toys | -.27 | .16 | [-.58, .05] | 2.73 | .099 |
| Child same-gender similarity | .24* | .08 | [.08, .40] | 8.27 | .004 |
| Child other-gender similarity | .12 | .08 | [-.05, .28] | 1.85 | .174 |
| Gender-typicality of parents’ career | .02 | .02 | [-.02, .06] | 0.77 | .381 |
| Gender-typicality of parents’ work hours | -.01 | .03 | [-.06. .04] | 0.21 | .647 |
| Gender-typicality of task division | -.08 | .04 | [-.16, .001] | 3.76 | .052 |
| Parent gender*Gender-typicality task division^2,4^ | .05 | .03 | [-.01, .11] | 2.97 | .085 |

^1^ Boys are reference category.

^2^ Fathers are reference category.

^3^ Mixed gender composition of children is the reference category.

^4^ The interaction between parent gender and gender-typicality of parents’ career and the interaction between parent gender and gender-typicality of work hours were not significant, and therefore deleted from the final model.

* *p* < .05
