# Supplementary material for: Children’s Views About Their Future Career and Family Involvement: Associations With Children’s Gender Schemas and Parents’ Involvement in Work and Family Roles
Source: Front Psychol. 2022 Jan 19;12:789764. doi: 10.3389/fpsyg.2021.789764 (PMC8809201; doi:10.3389/fpsyg.2021.789764)
Supplement: Supplementary file 2 [file Image_1.pdf]

Figure S1

Example Item Assessing Children's Expectations About Relative Future Involvement With Career Versus Family

Here are **Tom** and **Mike**. When they were your age, they played a lot together. Now they are all grown up.Now, **Tom** has job at a marketing company. He likes his job, although, he often has to stay there late and can't look after his kids. **Mike** used to work long hours too but now he only works 3 days a week so he can spend time with his family.

One day you will also be all grown up!

When you are grown up, who do you think you will be more like?

Picture of a man in a suit giving a presentation in an office	Picture of a man wearing an apron and is cooking in a kitchen
Tom	Mike

Note. Pictures are not actually displayed because of copyright, but are available upon request from the authors.
